# The Laboratorial Diagnosis of Dengue: Applications and Implications

**DOI:** 10.4103/0974-777X.52980

**Published:** 2009

**Authors:** Nina Rocha Dutra, Marília Barbosa de Paula, Michelle Dias de Oliveira, Leandro Licursi de Oliveira, Sérgio Oliveira De Paula

**Affiliations:** *Laboratory of Molecular Immunovirology, Department of General Biology, Federal University of Viçosa, Viçosa, Minas Gerais, Brazil*

**Keywords:** Clinical laboratory, Dengue, Diagnosis

## Abstract

The diagnosis of infection by the dengue virus relies, in most cases, on the clinical judgment of the patient, since only a few major centers have clinical laboratories that offer diagnostic tests to confirm the clinical impressions of an infection. At present, routine laboratory diagnosis is done by different kinds of testing. Among them are the methods of serological research, virus isolation, detection of viral antigens, and detection of viral genomes. The continued development of diagnostic tests, which are cheap, sensitive, specific, easy to perform, and capable of giving early diagnosis of the dengue virus infection is still a need. There are also other obstacles that are not specifically related to the technological development of diagnostic methods. For instance, infrastructure of the laboratories, the training of personnel, and the capacity of research of these laboratories are still limited in many parts of Brazil and the world, where dengue is endemic. Clinical laboratories, especially the ones that serve regions with a high incidence of dengue, should be aware of all the diagnostic methods available for routine these days, and choose the one that best suit their working conditions and populations served, in order to save lives.

## INTRODUCTION

Dengue is the most important arthropod-borne virus disease at the world level, and is very significant in the tropical and subtropical regions of the planet. It is caused by the infection with one of the four Dengue virus (DENV) serotypes, classified as DENV-1, DENV-2, DENV-3, and DENV-4. The DENV are RNA viruses, belonging to the *Flavivirus* genre of the *Flaviviridae* family, and their principal transmission vectors are arthropods of the *Aedes* genre, especially *Aedes aegypti*. The clinical manifestations of the infection vary from asymptomatic, passing by the classical dengue disease to more severe syndromes characterized by hemorrhagy and hipovolemic shock (hemorrhagic dengue syndrome).[1] Every year, it is estimated that infections by the Dengue viruses are responsible for more than 100.000.000 cases of the classical dengue disease and more than 500.000 cases of the hemorrhagic dengue syndrome all over the world. However, the real prevalence is not well known, since the notification in less developed countries is still considered unsatisfactory.[2]

In Brazil, according to the Department of Health Surveillance of the Ministry of Health, in the year 2008, only up to April, 230.829 suspect cases of dengue were notified, but only 3.298 were confirmed by laboratory tests. There were 77 deaths and 1.069 confirmed cases of DHF (dengue hemorrhagic fever), resulting in a lethality rate of 7.2% for DHF. There were also 3.298 notified cases of classical dengue disease with complications, resulting in 53 deaths. Data can be found on the website, www.portal.saude.gov.br. The number of reported cases of dengue and DHF in the Americas until October, 2008, is shown in Table 1 (Source: www.who.int).

**Table 1:** Number of reported cases of dengue and dengue hemorrhagic fever in the America by subregion, until October, 2008

	Dengue	DHF	Deaths
North America	0	0	0
Central America	65289	3273	4
Andean	56332	3830	8
Southern Cone	736381	9957	212
Hispanic Caribbean	3895	50	11
Caribbean	3800	21	3

Source: WHO, 2008

Aiming at the prevention and control of this disease progression worldwide, a committee formed by the members of the World Health Organization (WHO) had proposed practical guidelines, including the establishment of a surveillance system for dengue cases, in 1998, with the objective of achieving precise and early diagnosis and a quick reporting of the cases to the local public health authorities. To reach this goal, it is very important that the dengue diagnosis be fast and accurate, which is why every clinical laboratory should be aware of the most appropriate methods to do so, and adapt them in their daily routines, in order to serve the population in the best way.

Nowadays, in a majority of the countries in which DENV infections are most prevalent, most dengue diagnosis are still made based on the clinical judgment of the patients, since not every location counts on the existence of clinical laboratories for the confirmation of cases, and, what is even more of a concern, is that they do not count on the existence of clinical laboratories that are capable of making this kind of confirmatory test. It is important to be able to differentiate DENV infection from other infectious diseases that may require management with specific anti-microbial therapy.[3] In consequence of this fact, the objective of this study is to make a survey of the methodologies currently available for laboratorial dengue diagnosis, aiming to help clinical analysts all over the world to recognize the most appropriate ways to do that in their laboratories, routinely.

## CLINICAL AND LABORATORIAL MANIFESTATIONS

The clinical features of the DENV infections vary from the absence of symptoms, passing by mild fevers similar to a cold (classical dengue disease) until more severe cases, with hemorrhagic tendencies. This clinical variability is still not fully understood, and seems to be related to the age and genetics of each individual, but mostly to the patient's immunological and nutritional conditions.[4]

The incubation period after the inoculation of the virus by the bite of the infected vector is four days on an average. The disease may be manifested with fever and petechiae, and in these situations, the clinical differentiation from other viral diseases may not be possible. The recovery is usually quick. In more severe cases, the body temperature rises quickly (sometimes reaching over 39°C) and may persist for five or six days.[5] Other symptoms may appear, such as, headache, retrorbitary pain, arthralgia, myalgia, red spots on the skin, hepatomegaly, and abdominal pain. In these cases, the laboratorial parameters are usually normal, except for the platelets count, which may be slightly decreased (around 100.000/mm^3^), and for the liver enzymes in the blood serum (alanine aminotransferase in particular), which may be somewhat increased, but rarely trespassing the level of 100 UI/mL.[6] The recovery can be reached in seven to 10 days.

In more severe outcomes of the infection by Dengue viruses (the dengue hemorrhagic syndrome), the first symptoms are very similar to the classic disease just described. The first hemorrhagic manifestations usually show up only around the third day of infection, and they consist mainly of sparse petechiae all over the body. These are usually associated with a positive tourniquet test, and sometimes the tourniquet test is already positive even before the outcome of the petechiae. What makes this test very important in the early detection of this hemorrhagic syndrome.[7] There may also be bleeding in the venopunction site, gastrointestinal tract, nose, and gums. After two to seven days, when the fever starts to decrease, the patient is commonly found exhausted and with cold sweating in the body extremities. With appropriate treatment, this phase may resolve in one or two days. If not, the patient may evolve to shock as a result of plasma leakage to the extravascular compartment and disseminated intravascular coagulation.[4] The laboratorial investigation of hemorrhagic dengue reveals that thrombocytopenia may reach under 20.000 platelets/mm^3^, hemoconcentration with hematocrits are elevated up to 20% or more, hypoalbuminemia, and moderate elevation in the dosage of serum aminotransferases and urea. The partial thromboplastin time (PTT) and thrombin time (TT) may be extended. The levels of fibrinogen and complement proteins are often found to be decreased and correlate with the severity of the disease.[8]

## DENGUE LABORATORY DIAGNOSIS ACTUAL OVERVIEW AND PERSPECTIVES

The accurate and efficient laboratorial diagnosis of the infections by the Dengue viruses is of great importance to the clinical care of the patient, the epidemiological surveillance, in the study of the pathogenesis of this infection, the research of vaccine formulations, and, furthermore, it also contributes to the early detection of epidemic foci, providing useful information to health authorities in time, to carry out the localization and contention on the dissemination of the virus. The use of laboratorial diagnostic tools suitable for the detection of infection by the Dengue viruses is critical for the determination of important features, such as, the actual number of fatal cases, the viral strain involved in a special disease focus, and the estimate of the total incidences during an epidemic.

Nowadays, the laboratorial diagnosis of dengue can be made following different research lines, which are: virus isolation, detection of viral genome, detection of viral antigens, and serological studies. Actually, serology is the most widely used tool in the laboratory routine.[9] Obviously, the clinical, geographic, and epidemiological data about the patient are very important when evaluating the results of laboratorial research.

## SEROLOGICAL RESEARCH

The infection of a susceptible (nonimmune) individual by the DENV produces a primary response characterized by the slow development of low titers of antibodies (Ab). The first isotype of Ab to be detected is the anti-Dengue IgM. The second one, anti-Dengue IgG, appears in low titers around the end of the first week of disease installation, and these titers may rise slowly.[10] In case of a secondary infection (meaning, DENV infection on a previously immunized individual with other serotype of DENV or another flavivirus, since, according to Vázquez, in 2007,[11] the antibodies to these viruses present cross reaction) the antibody titers rise quickly, and these Ab are able to react widely with different flaviviruses. High titers of IgG are detectable even in the acute phase of the disease and they keep rising for the following two weeks approximately. The kinetics of the anti-Dengue IgM titers in the secondary infection by DENV is more variable, appearing late, during the febrile phase of the illness, often preceded by the IgG. Some false-negative anti-Dengue IgM reactions may be observed in secondary infections. However, even detection of anti-Dengue IgM is not useful for serotyping of the virus, due to the cross reactivity that has presented.[9]

According to Chadwick *et al.,*[12], clinically speaking, diagnostic seroconversion is defined by the rise (or fall) of the anti-Dengue antibodies in at least 4x, measured in two serum samples by hemagglutination inhibition (HI), Complement fixation reaction (CFR), plaque reduction neutralization technique (PRNT), or enzymatic immunosorbent assays (ELISA). Due to the antigens that are shared by all flaviviruses and that produce cross reactivity, virus-specific laboratory diagnosis is not possible using these techniques, except for the PRNT, which presents the highest specificity for the determination of anti-Dengue antibodies among these presented assays.

With the objective of determining the presence and quantity of anti-Dengue neutralizing antibodies, many protocols have been developed. Cultures of VERO and BHK-21 cells are often utilized. In the actual days, only a few laboratories use the PRNT assay in their daily routines.

The hemagglutination inhibition (HI) is the serological technique most widely accepted; however, since it is time-consuming, ELISA has become the technique that is most often applied to the serological research of DENV infection.[12]

The anti-Dengue IgG detection by ELISA is largely utilized for the classification of cases, based on the type of infection, primary or secondary. Some protocols use serial serum dilutions to do the anti-Dengue antibodies titration. In others, an IgM/IgG ratio higher than 1:78 is considered indicative of primary infection, while the ratio under this cut-off number would be indicative of secondary infection.[9]

The utility of the detection of anti-Dengue IgA as a recent infection indicator has already been demonstrated by some researchers. Talarmin *et al.*[13] have determined the presence of anti-Dengue IgM and IgA antibodies in the sera of 178 patients with classic dengue disease. IgA antibodies were detected from the sixth to the twenty-fifth day after the onset of fever. Groen *et al.*[14] also have suggested the diagnostic value of anti-dengue IgA detection in the serum using immunofluorescence assays, even though the highest percentage of IgA detection was observed in acute phase serum samples of secondary infections.

The detection of anti-Dengue IgM antibodies utilizing ELISA represents one of the biggest advances and has become a valuable tool in dengue laboratory routine diagnosis. More specifically, the ELISA IgM-capture technique (MAC-ELISA), which is based on the specific detection of these antibodies in the serum through their capture using anti-IgM human antibodies previously adsorbed to the solid phase, have presented only around 10% false-negative reactions and 1.7% false-positive reactions.[9,15]

In 2007, Kumarasamy *et al*.[16] evaluated a MAC-ELISA assay to the NS1 protein of the DENV (PLATELIA™ DENGUE NS1 AG test kit, BIO-RAD, France), with the objective of demonstrating its potential application in the early laboratorial diagnosis of the infection by these viruses. This group obtained a general sensitivity of 93.4% and specificity of 100%, out of 354 samples, 213 of them being from acute infections. The technique was compared with the viral isolation in cell cultures and with the RT-PCR assay, and achieved great results, along with the use of ELISA for the detection of anti-NS1 IgG (Panbio Dengue IgG Capture ELISA, Australia).[16] Recent studies have shown that the Dengue virus nonstructural 1 (NS1) antigen, a highly conserved glycoprotein produced in both membrane-associated and secreted forms and abundant in the serum of patients in the early stages of infection, may be an appropriate marker of acute Dengue virus infection.[17]

Clinical laboratories all over the world can use diverse diagnostic kits already available in the market by different suppliers, similar to the ones that are shown in Table 2.

**Table 2:** Kits available in the market for the detection of anti-dengue antibodies[9]

Commercial kits	Detected Ig isotypes	Format	Sensitivity (%)	Specificity (%)
PanBio dengue Duo	IgM/IgG	ELISA	94[31]	100 approximately[31]
Dengue duo Rapid dtrip test, PanBio	IgM/IgG	Immunochromatographic test	76–100 for IgM detection [32] 88–94 for IgG detection[32]	88–99[32]
MRL diagnostic dengue	IgM	ELISA	97,8[33]	100% approximately[33]
Blot IgMTM, diagnostic biotechnology ltd.	IgM	Immunoblot	96,9[34]	87,7[34]
Venture technologies dengue IgM and IgG dot blot	IgM/IgG	Immunoblot	100[35]	97[35]
Integrated diagnostics	IgM	Immunochromatographic test	92,6[32]	94,3[32]
UMELISA Dengue IgM	IgM	Ultramicro-ELISA	99,4[36]	94,8[36]
PanBio dengue IgG dapture ELISA	IgG anti-NS1	ELISA	95[16]	94[16]
PLATELIA™ Dengue NS1 ag, BIO-RAD	IgM anti-NS1	MAC-ELISA	93,4[16]	100[16]

Modified from Guzmán and Kouri[9]

## VIRAL ISOLATION

DENV viremia is short, usually observed two or three days before the onset of fever until five days after that, at most. In a previous study, our group had determined that at most, biological samples for viral isolation should be collected until the fourth or fifth day after the establishment of the disease.[18]

The DENV are heat sensitive, so care should be taken while manipulating samples destined to viral isolation, either relating to the processing itself or relating to the time of delivery of the sample to the laboratory. The sample can be stored for short periods of time at 4°C, but, for longer storage periods, it should be kept at −70°C.[19]

The inoculation of samples in mosquitoes (vectors) is the most sensitive system for the DENV isolation. Larvae can be used as well as adult insects. In general, the *Toxorhynchites* mosquitoes are preferred, since they are bigger than the *Aedes* and they are not hematophagous. However, adult male *A.aegypti* and *A.albopictus* are also used. The viral inoculation practice in mosquitoes demands a lot of technical expertise, and most times, it is preferable to do the isolation in cell cultures for routine laboratorial diagnosis. The cell lines utilized are also from mosquitoes, and have been shown as very efficient in viral isolation. The cell line C3/36, from *A.albopictus*, is the choice cell line for routine isolation of DENV, although the AP61 cell line from *A.pseudocutellaris,* has also been used with success.

The oldest and less sensitive method for DENV isolation is the inoculation into the brain of new-born mice, which actually is used only when no other method is available. Although many animals develop symptoms of encephalitis, the majority does not show any sign of disease after the procedure.[9]

The identification of the isolated viral strain is generally done by immunofluorescence techniques using monoclonal anti-Dengue serotype-specific antibodies on cells, in a culture. Usually, the samples pass through a preliminary trial with polyclonal anti-Dengue antibodies, and the positives are confirmed with the monoclonal antibodies specific to each one of the four DENV serotypes. Some strains are not easily identified due to the low viral concentrations in samples. Some researchers recommend the passage of one or more samples in the cell culture systems, to augment the viral concentration.[20]

According to Kao *et al.,*[21] of late, the flow citometry has also been shown to be a useful method for DENV-1 identification, allowing the detection of the virus 10 hours before the results, with immunofluorescence, using anti-NS1 monoclonal antibodies.

## DETECTION OF VIRAL ANTIGENS

In the past few years, some very sensitive viral antigen detection systems have been standardized in the ELISA format. In 1995, Malergue and Chunge[22] applied a fluorogenic ELISA amplified with streptavidin and biotin for the detection and identification of DENV-3 antigens in the patients´ sera. The method showed 90% sensitivity and 98% specificity when compared to the viral isolation in C6/36 cells.

In 1997, Kittigul *et al*.[23] demonstrated that DENV antigens could be detected in higher frequencies in mononuclear peripheral blood cells when compared to serum (53.8% and 18.9%, respectively), also utilizing an ELISA streptavidin-biotin system.

A commercial kit based on two ELISA systems, one for the detection of antigens (“blue kit”) and the other for viral identification (“red kit”) is already available in market. According to the manufacturer, the “blue kit” reaches 84% sensitivity and 89% specificity, while the “red kit” reaches 91% sensitivity and 93% specificity (GLOBIO BLUE AND RED KIT for antigen detection, Globio Corp. Beverly, MA, USA).

Immunohistochemical techniques using peroxidase or alkalin phosphatase markers have also been pointed out to be as useful, in the detection of DENV antigens in tissue samples included in paraffin and fixed in formalin, even though this technology is not widely applied to the laboratorial diagnosis in endemic countries.[6]

## VIRAL GENOME DETECTION

The polymerase chain reaction (PCR) has become a very important tool in the diagnosis of dengue and many other viral diseases, as well as for the epidemiological surveillance of the efficiency studies of new vaccine candidates and antiviral drugs. In case of DENV (and all the other RNA viruses), DNA amplification is preceded by a reverse transcription reaction for the production of a complementary DNA (cDNA) to the viral genomic RNA. A great number of PCR protocols have already been standardized, many of them involving a combination of primers, which are simultaneously specific to each of the four DENV serotypes. These primers may anneal in different regions of the viral cDNA, for example, in the regions of NS1, E, prM, and NS5 genes. Some protocols are able to detect very low viral copy numbers, such as 50–100 copies/mm.[6,24]

When applied appropriately, PCR presents considerable advantages as a dengue diagnostic tool. The use of PCR allows DENV detection in long-term storage samples,[25] as well as in entomological surveillance, in other words, the control of the mosquitoes species that are participating in the viral transmission.[26] It also allows the identification of the serotypes responsible for a determined infection focus[27] and the study of the genetic diversity of the strains, in order to identify the origins of epidemics and reveal virulence markers, when helped by nucleotide sequencing. That makes possible the classification of DENV serotypes into subtypes according to genotypes, as exemplified by Guzmán and Kouri, in 2004[9] [Table 3].

**Table 3:** Rank in genotypic subtypes

Serotype DENV	Subtype
DENV-1	I French Polynesia/Fiji/Singapore/Indonésia/Nauru/New Caledonia/Tonga; II Jamaica/French Guyana/New Caledonia/Brazil/México/Aruba/Cuba/Peru/Nicarágua/Thailand/Senegal/Malaysia/Puerto Rico; III Philippines/Thailand
DENV-2	I Puerto Rico/Tahiti/Tonga/Colombia/Mexico/Venezuela/Trinidad; II Taiwan/Philippines/New Guinea/Thailand; III Vietnam/Thailand/Jamaica; IV Indonesia/Seichelles/Burkina Fasos/Sri Lanka; V Ivory Coast/Burkina Faso/Senegal
DENV-3	I Philippines/Malaysia/Indonesia/Tahiti/Fiji; II Thailand; III Sri Lanka/Samoa/India/Mozambique; IV Puerto Rico/Tahiti
DENV-4	I Thailand/Philippines/Sri Lanka; II Tahiti/Puerto Rico/Brazil/New Caledonia/El Salvador/México/Dominica/Indonesia

Modified from Guzmán and Kouri[9]

Finally, new PCR protocols and methodologies have been showing up, and they permit quick detection and quantification of viral RNA in samples. These protocols are based widely in the RT-PCR technology, that is, PCR preceded by a reverse transcription in a one-step reaction, as it has already been described by our group, in a work published in 2002,[28] and by many other researchers, among them Barkham *et al.,*[24] Chutinimitkul *et al.,*[25] Kumaria *et al.,*[19] Prado *et al.,*[26] and Lemmer *et al.*[27]

## BIOLOGICAL SAMPLES FOR THE LABORATORIAL DENGUE DIAGNOSIS

Serum is the sample of choice for serological techniques, although other types of samples such as blood collected in filter paper, urine, and saliva have already been utilized in anti-Dengue IgM detection, if they were collected in the appropriate period (after five days of onset of fever).[9] In a previous work, Mizuno *el al*., has also demonstrated the detection of anti-Dengue IgM antibodies in the saliva of 65.8% out of 38 patients infected with DENV, with a higher positivity (> 80%) in the samples collected after five days of fever onset.[29]

With regard to the viral isolation methodologies, serum is once again the sample of choice for the diagnosis routine, even though DENV may also be detected in plasma, leukocytes, and biopsy (or necropsy) tissues, such as, those of lungs, liver, lymph nodes, and thymus. The samples should be sent to the laboratories as quickly as possible, due to the DENV sensitivity to heat.

As for the viral antigen detection techniques, serum and plasma have been utilized, and as mentioned before, Kittigul *et al.*[23] have also demonstrated the possibility of detection of these antigens in peripheral blood mononuclear cells.

The viral genomic RNA has also been shown by PCR in serum, plasma, infected culture cells, infected larvae, and adult mosquitoes collected in the field, and fresh tissues or tissues fixed in formalin and included in paraffin. Mizuno *et al.*[29] described a case in 2007, where they were able to successfully detect DENV-1 genome in the urine and saliva of a patient infected in Japan, but not in the plasma samples obtained from the same patient, using the RT-PCR method. Prado *et al.*[26] have also been able to detect the viral genome in whole blood samples collected in filter paper up until nine weeks after collection, the samples being stored at room temperature as well as at 4°C and −70°C.

In 2002, our research group tested whole blood, serum, and buffy coat samples from 75 IgM-positive patients for the detection of DENV genome, using the RT-PCR method. We obtained higher positivity in the serum samples (14 out of 17 positive samples were serum samples).[18]

## FINAL CONSIDERATIONS

The IgM-capture ELISA, viral isolation in mosquito cell lines, PCR, and DENV-specific monoclonal antibody techniques represent the biggest advances in laboratory methods for dengue diagnosis.[9] However, some obstacles are still observed in developing new laboratory tools for dengue diagnosis.

Viral isolation in the cell culture is a time-consuming process that reproduces viral replication in the host's cells, and for this reason, may not be the most appropriate method to be used as the identification routine for acute infections. The PCR techniques demand specific laboratory equipment and suitable physical structure, in addition to an extensive evaluation of protocols under the conditions of the field which the laboratory meets, since there may be differences between strains circulating in different places. IgM detection requires appropriate collection time and its results may be confused with false-positive reactions (since anti-DENV IgM shows extensive cross-reaction) and by the prolonged presence of these antibodies in some people's blood. Thus, some commercial kits still need to be critically evaluated as to their results, cost, and feasibility of reagents.

The continued development of inexpensive diagnostic tests - sensitive, specific, and easy to perform, and which are able to provide early detection of dengue virus infection - is still a need. The following items show the aspects that need more attention:

- Development of tests for early diagnosis in individuals.
- Development of serological tests capable of differentiating the infection by DENV from infections by other flaviviruses, and more specifically, to differentiate between the serotypes of DENV.
- Development of protocols, which are easy and inexpensive for the characterization of genomic and viral load, including protocols for use in the field.
- Modification of the existing protocols, to simplify the transport and handling of biological samples.
- Use of recombinant antigens as components of diagnostic tests and as tools to evaluate these tests.
- Development of laboratory tools that might suggest a prognosis, allowing better monitoring of clinical cases. Beyond these specific items, researchers must also direct attention to the optimization of mechanisms aimed at giving greater viability of reagents (antigens, monoclonal antibodies, cell cultures, positive and negative controls, etc.), as well as to the optimization of methods for the standardization of diagnostic protocols in endemic areas, to the quality control and the sharing of information and experience between endemic areas, including the development of research projects in collaboration with laboratories.

There are still some problems and needs that are not specifically related to the technological development of diagnostic methods. For example, the infrastructure of the laboratories, training of technical staff, and research ability is still limited in many parts of Brazil and the world, where dengue is endemic. These factors negatively influence epidemiological surveillance, the monitoring of clinical cases, and the development of new approaches to the control of dengue. It is an urgent need to mobilize funds from the government to improve the ability of different public health services, the infrastructure of laboratories, hospitals, and basic care units, among others, in addition to training personnel; thus generating a better control and prevention of this disease.

The problem with dengue is present in reality, and therefore, clinical laboratories should be interested in all the diagnostic methods available for routine, and determine which best fits their working conditions and the population served, thus providing allowance for health professionals to intervene on both a curative and preventive front, by saving lives.
